# Access to quality diagnosis and rational treatment for tuberculosis: real-world evidence from China–Gates Tuberculosis Control Project Phase III

**DOI:** 10.1186/s40249-021-00875-8

**Published:** 2021-06-29

**Authors:** Zhi-Peng Li, Wen-Hui Mao, Fei Huang, Ni Wang, Li-Ping Ma, Li-Qun Zhang, Meng-Qiu Gao, Wei-Bing Wang, Qi Zhao, Sheng-Lan Tang

**Affiliations:** 1 School of Public Health, Fudan University, 138 Yixueyuan Road, Shanghai, 20032 China; 2 Duke Global Health Institute, Duke University, 310 Trent Drive, Durham, NC 27710 USA; 3 Chinese Center for Disease Control and Prevention, Beijing, China; 4 Beijing Chest Hospital, 97 Machang, Tongzhou, Beijing, China

**Keywords:** Tuberculosis, Evaluation, Diagnosis, Treatment, Rapid molecular test, Second line drugs

## Abstract

**Background:**

China has successfully reduced tuberculosis (TB) incidence rate over the past three decades, however, challenges remain in improving the quality of TB diagnosis and treatment. In this paper, we assess the effects of the implementation of “China National Health Commission (NHC) and Gates Foundation TB Prevention and Control Project” on the quality of TB care in the three provinces.

**Methods:**

We conducted the baseline study in 2016 and the final evaluations in 2019 in the 12 selected project counties. We obtained TB patients’ information from the TB Information Management System and reviewed medical records of TB cases in the TB designated hospitals. We compared TB diagnosis and treatment services with the national practice guideline and used Student’s *t-*test and Pearson *χ*^2^ tests or Fisher’s exact tests to compare the difference before and after the project implementation.

**Results:**

The percentage of sputum smear-negative (SS–) patients taking culture or rapid molecular test (RMT) doubled between 2015 and 2018 (from 35% to 87%), and the percentage of bacteriologically confirmed pulmonary TB cases increased from 36% to 52%. RMT has been widely used and contributed an additional 20% of bacteriologically confirmed TB cases in 2018. The percentage of TB patients taking drug susceptibility tests (DST) also doubled (from 40% in 2015 to 82% in 2018), and the proportion of TB patients receiving adequate diagnosis services increased from 85% to 96%. Among all SS– TB patients, over 86% received the recommended diagnostic services at the end of the study period, an improvement from 75% prior to the project implementation. However, the proportion of TB patients treated irrationally using second-line anti-TB drugs (SLDs) increased from 12.6% in 2015 to 19.9% in 2018. The regional disparities remained within the project provinces, albeit the gaps between them narrowed down for almost all indicators.

**Conclusions:**

The quality of TB diagnosis services has been improved substantially, which is attributable to the coverage of new diagnosis technology. However, irrational use of SLDs remains a concern after the project implementation.

**Graphic abstract:**

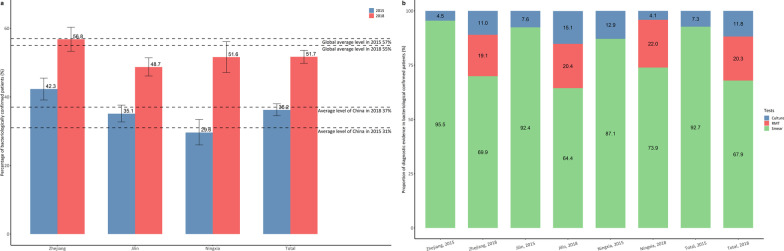

**Supplementary Information:**

The online version contains supplementary material available at 10.1186/s40249-021-00875-8.

## Background

Tuberculosis (TB) remains a global health challenge. In 2017, TB caused the highest number of deaths and the second highest disability-adjusted life years (DALYs) among all communicable diseases globally. Combating TB has been prioritized by the Sustainable Development Goals by 2030. The World Health Organization (WHO) End TB Strategy endeavors to reduce TB deaths by 95% and eliminate catastrophic health expenditures caused by TB by 2035 [1, 2]. China has one of the highest burdens of TB and remarkable achievements have been made to improve the control and care for TB [3] between 2000 and 2010. The directly observed treatment strategy (DOTS) has been scaled up across the country by the Chinese Center for Disease Control and Prevention (China CDC), and significant government subsidies have been provided to enable free first-line anti-TB medicines, smear tests and chest X-rays [4, 5]. These efforts have led to significant declines in the incidence of TB in China [3].

TB clinical care has been shifted from the CDCs to TB designated hospitals since 2010. Despite the integrated TB care model has been scale-up, the diagnosis and rational treatment of TB remain key issues of concern. In 2018, only 37% of the TB notified cases in China were confirmed through bacteriological detection, much lower than the global average of 55% [6]. Bacteriological detection of TB is essential for timely and precise diagnosis of TB and early initiation of treatment [7, 8]. Yet the majority of TB patients in China are diagnosed clinically based on symptoms, chest radiography, suggestive histology and immunologic tests that are generally associated with low specificity for TB diagnosis. This could potentially delay treatment for TB or in contrast, lead to unnecessary treatment for those misdiagnosed.

China has also seen growth in the prevalence of multidrug-resistant TB (MDR-TB) or rifampicin-resistant TB (RR-TB) over time. In 2018, it had an estimated 66 000 MDR/RR-TB incidents, among which only 22.8% were laboratory-confirmed, presenting a much lower MDR-TB detection rate compared to the global average of 39% in the same year [6]. The low detection rate of drug-resistant TB is partly attributable to the poor access to the drug susceptibility test (DST), which can lead to a low treatment rate and further spread of MDR/RR-TB [9].

A recent study reported that over- and under-treatment of TB co-existed in China [10]. Despite the first-line anti-TB medicines is free of charge to the patients, irrational use of anti-TB medicines, especially second-line anti-TB drugs and the use of other ancillary drugs (e.g. liver protection drugs) have been commonly reported [11–13]. On the other hand, follow-up examinations during the treatment that include routine blood tests, liver and renal function tests, and among others were not properly conducted according to the clinical practice guideline.

Several studies have evaluated the significance of rapid molecular test (RMT) and proved that it has substantially improved the quality of TB diagnosis, with better sensitivity and specificity than traditional tests. Additionally, the testing time of RMT is shorter, resulting in more timely initiation of treatment for TB patients [14, 15]. Despite this evidence, RMT has not widely applied for TB diagnosis in China.

The China National Health Commission and China CDC established a partnership with the Bill & Melinda Gates Foundation (BMGF) to strengthen TB control in China. Strengthening the capacity of TB care delivery is one of the components of the China National Health Commission and the Bill & Melinda Gates Foundation TB prevention and control project (China-Gates TB project), Phase III. The relevant interventions include promoting the use of RMT and providing training on TB diagnosis and clinic treatment in the TB designated hospitals. The phase III project was initiated in 2016 for a three-year implementation in the three provinces: Zhejiang, Jilin, Ningxia.

The purpose of this study was to evaluate the impact of China-Gates TB project Phase III on access to quality diagnosis and rational treatment of TB from a population perspective. Specific objectives are to evaluate whether the implementation of the project: (i) has improved the accessibility to culture or RMT services for sputum smear-negative (SS–) patients and increased the proportion of bacteriologically confirmed TB case; (ii) has increased the DST coverage rate for bacteriologically confirmed TB patients; (iii) has increased the proportion of TB patients receiving adequate diagnostic services; (iv) has reduced the proportion of TB patients receiving secondly line drugs (SLDs) irrationally; and (v) has increased the proportion of TB patients receiving recommended follow-up examinations. Finally, we indicated policy implications based on the experience learned from the project implementation for the continued improvement of TB detection and treatment in China.

## Methods

### Study design and setting

The China-Gates TB project, Phase III was implemented in three provinces/autonomous region in China: Zhejiang, Jilin, and Ningxia. These provinces represent high, middle and low level of economic development. We present the demographic and socioeconomic characteristics of each province in Additional file 1: Appendix 1.

#### Major interventions implemented in the project sites

The project implemented a comprehensive package of interventions in December 2016 to improve TB care and control. The interventions include: (1) Strengthening TB suspect screening and referral system; (2) Expanding the use of new TB diagnostic technologies such as RMT; (3) Training on rational treatment regimens based on DST results; (4) Strengthening public health functions of TB designated hospitals; (5) Improving collaboration between CDC, TB hospitals and primary health clinics; (6) Building an e-learning system and implementing other capacity-building activities on TB diagnosis and clinical treatment; and (7) Developing the financing mechanism to reduce patients’ financial burden in seeking care for TB. Table 1 highlighted the details of major interventions for improving the quality of clinical service.

**Table 1:** Three major interventions implemented in project sites during 2015–2018

Intervention area	Before (2015)	After (2018)
Access to health services for SS– patients	No requirement	All the SS– patients receive culture or RMT
DST coverage for TB patients	DST screening for MDR-TB patients amongst high-risk TB patients	All the bacteriologically confirmed TB patients should receive DST
TB treatment regimens	Using rational treatment regimens without DST results	Use rational treatment regimens based on DST results

*SS–* sputum smear-negative, *DST* drug susceptibility test, *TB* tuberculosis, *MDR-TB* multidrug-resistant tuberculosis, *RMT* rapid molecular tests

#### Selection criteria for study sites

With the consideration of the different levels of socio-economic development, we selected two prefectures from each of the three provinces, one with higher gross domestic products (GDP) per capita in 2015 and one with lower GDP per capita. In the selected prefecture, we used the following selection criteria to choose study counties, in total 12 counties involved in the evaluation:

- A county with higher GDP per capita and a county with lower GDP per capita.
- No less than 200 cases of pulmonary tuberculosis patients registered every year in each county. If no county meets the standard, select the county with the largest number of patients registered.
- Counties in which comprehensive mode of intervention was launched in 2017.
- Cover plain and mountainous areas.

### Data collection

We performed routine data collection and conducted medical records review in July 2016 and July–August 2019 in our study sites. Patients with pulmonary tuberculosis diagnosis were eligible for analysis.

#### Routine data collection

We used the TB Information Management System (TBIMS), a routine registration data operated by CDCs across China, to obtain TB and DRTB patient information for those registered between January 1–December 31, 2015 and January 1–December 31, 2018 in the study sites.

From TBIMS, we extracted the following information: demographic information such as gender, age and registered location of the patient, diagnosis, bacteriological testing results including sputum smear, sputum culture, RMT, DST, etc. Patients with diagnosis of extrapulmonary tuberculosis or tuberculous pleurisy were excluded from analysis. For the baseline analysis for data from 2015, 2904 TB patients were eligible and included in the analysis. For the final evaluation in 2018, 2606 TB patients were eligible and included in the analysis.

#### Medical records review

We conducted medical records review in our study sites for the baseline study in 2016 and the final evaluation in 2019. We used the same checklist developed by TB clinical experts and public health experts to review medical records. The checklist included information about demographics, symptoms, diagnosis (including sputum smear, sputum culture, RMT, Chest X-ray and CT examinations, immunological examinations, differential diagnosis information), treatment regimens and outpatient and inpatient records. Please refer to Additional file 1: Appendix 2 for details about the checklist. We reviewed medical records for pulmonary TB patients diagnosed between January 1ؘ–December 31, 2015 and January 1–December 31, 2018. Patients with diagnosis of extrapulmonary tuberculosis or tuberculous pleurisy were excluded from the analysis.

*(1) Process of medical records review*

According to our inclusion and exclusion criteria, staff from TB designated hospitals in the study sites extracted the eligible medical records, starting with the records with the most recent date, until they reached the required sample size. A medical doctor specialized in TB and a research assistant who trained before the study from the evaluation team reviewed the medical records and completed the checklist for each patient.

*(2) Sample size*

The number of eligible TB patients varied by the study county. We collected at least 30 TB patients from each county from the TB case registration in 2015. The sample size of the final evaluation in 2018 was determined by the rational diagnosis and treatment rate of the baseline survey as following: (1) outpatient medical records: 100 outpatient records of those who completed TB treatment from each county were reviewed to assess whether the patients have received adequate diagnostic; (2) inpatient medical records: 55 SS– TB patients’ records from each county were reviewed to assess the quality of diagnosis for TB patients without bacteriological confirmation; 40 active TB patients’ records including both sputum smear-positive (SS +) and SS– to assess the usage of second-line anti-TB medicines.

In counties where the number of eligible TB patients is smaller than the required sample size, all eligible medical records were reviewed. In total, we reviewed 829 records in 2015 and 1185 records of TB patients in 2018. We reviewed 235 and 356 inpatient medical records for TB patients without bacteriological confirmation, respectively in 2015 and 2018; and we reviewed 286 and 690 eligible medical records of TB inpatients from 2015 and 2018, respectively (Please refer to Additional file 1: Appendix 3 for more information about sampling).

### Measurements and definitions

We adopted measurements for the access to quality of TB diagnosis and treatment based on the indicators recommended by the WHO TB Report [16] and the national TB practice guideline in China.

#### Access to quality of TB diagnosis

According to the 2008 Guideline for the Implementation of Tuberculosis Control Programme in China, the quality of TB diagnosis was defined as patients who receive proper diagnostic test(s) for TB including the sputum culture and RMT, and other examinations such as Chest X-ray or CT examinations, and immunological examinations [17]. We used the TBIMS data to collect information about the different types of TB diagnostic tests that were performed among SS– patients and bacteriologically confirmed TB cases and coverage of DST for TB patients. Then we compared the information we collected with the national practice guideline to assess the proportion of TB patients who received appropriate tests. Proper use of DST reflected the quality of the diagnosis. We included the following dimensions for the quality of the diagnosis.

1. Diagnosis for smear-negative TB patients: Among all SS– TB patients, we reported the percentage of the patients who have further received either culture or RMT or both tests. (Source: TBMIS).
2. Diagnosis for bacteriologically confirmed TB patients: Bacteriologically confirmed TB case is defined as patients from whom a biological specimen is positive by either smear microscopy, culture or rapid molecular tests (RMT) such as the Xpert MTB/RIF® assay. We report the proportion of bacteriologically confirmed TB cases. Then we report the share of smear microscopy positive, culture positive and RMT positive among bacteriologically confirmed TB cases (Source: TBMIS).
3. Diagnosis for drug susceptibility: Diagnosis for drug susceptibility was measured by drug susceptibility testing (DST) coverage for bacteriologically confirmed TB patients. We report the proportion of bacteriologically confirmed TB cases tested for drug resistance by phenotypic DST or RMT. Then we present a share of RMT and phenotypic DST among bacteriologically confirmed TB cases (Source: TBMIS).
4. Adequate diagnostic services among TB patients: According to the national practice guideline, we developed two indicators to assess the proportion of TB patients who have received adequate diagnostic services.

TB patients should receive at least one sputum examinations (sputum smears or sputum culture) and at least one of the Chest X-ray or CT examinations. Among all reviewed outpatient medical records of TB patients, we reported the percentage of TB patients who received these recommended tests (Source: medical record review). TB patients without bacteriological confirmation are recommended to receive all of the following tests: (a) three sputum smears with negative results; (b) chest X-ray or CT examinations showed lesions consistent with active tuberculosis, and (c) one of the following: typical TB symptoms; a positive result of immunological tests from tuberculosis skin test (PPD), anti-tuberculosis antibody test or interferon-gamma release assays (IGRAs); differential diagnosis to rule out other diseases including tumor marker test, exfoliated cell examination, bronchoscopy, lung biopsy and general antibiotics treatment. Among all reviewed inpatient medical records of TB patients, we reported the percentage of TB patients who received these recommended tests, breaking them down into asymptomatic pulmonary TB patients and symptomatic pulmonary TB patients, respectively. Suspicious TB symptoms include cough, expectoration, hemoptysis and fever. Proportions of different types of diagnostic tests used by TB patients without bacteriological confirmation can be found in Additional file 1: Appendix 4 (Source: medical record review).

#### Rational prescriptions of clinical services

We collected data from medical records on two indicators to determine whether or not a patient received rational clinical services. Specifically, our analysis focused on the irrational prescription of second-line anti-TB drugs, and the follow-up examinations during the whole treatment course.

1. *Proportion of TB patients treated with irrational second-line drugs (SLDs)* Among TB patients with inpatient medical records, a proportion of TB patients received irrational second-line anti-TB drugs during treatment. Irrational SLDs treatment was defined by (1) using SLDs as an anti-inflammatory treatment before obtaining drug susceptibility testing results; (2) using SLDs to replace pyrazinamide without specifications (Source: medical record review).
2. *Proportion of TB patients receiving recommended follow-up examinations during treatment course* According to the national practice guideline, the following examinations (with specific time frames) that mainly to monitor the adverse reactions of TB treatment have been analyzed: blood routine test and routine urine test before treatment and one-month completion of treatment; liver and renal function test before treatment, each month during enhanced treatment, and whenever a patient has nausea, vomiting or liver pain [17]. We report a proportion of TB patients receiving recommended follow-up examinations during the treatment course, proportions of patients receiving the recommended routine blood tests, liver and renal function test, and routine urine test, respectively. Specifically, seven follow-up tests during the treatment course indicated properly follow-ups while three tests were the minimal requirements (results presented in Additional file 1: Appendix 3). Proportions of different types of tests used by TB patients can be found in Additional file 1: Appendix 3 (Source: medical record review).

### Data analysis

We used a before-and-after approach to assess the changes in access to quality of diagnosis and treatment for TB in all study sites. All indicators were assessed for years 2015 (baseline) and 2018 (end line). We also compared the national average level and global level of the selected indicators when publicly available. We compared the changes between 2015 and 2018 by using Student’s *t-*test on continuous variables and Pearson *χ*^2^ tests or Fisher’s exact tests on categorical variables. We used two-sided tests with 95% confidence interval (CI) for all analyses. Data were double entered by using EpiData 3.1 (The EpiData Association, Odense, Denmark). Statistical analyses were done with SPSS22.0 (International Business Machines Corporation, Armonk, New York, USA).

### Quality assurance

Multiple approaches were taken to ensure quality assurance in both study design and data collection. We hosted three rounds of expert consultation meetings to determine both the plan for data collection and the design of the checklist for the review of medical records. Experts included clinical and public health experts in TB diagnosis and treatment in China, staff from the TB management unit of China CDC and local CDC, and international experts from academic institutions. All of the data collection instruments, tools and procedures were tested before the investigation began. Investigators engaged in the data collection received proper training from clinical and public health experts in TB diagnosis and treatment. All data were cleaned after collection and underwent logical checks for accuracy and consistency.

### Ethical considerations

The project evaluation protocol was reviewed and approved by the Institutional Review Board at the Duke University (#D0877 and #2017-0768). The China-Gates TB project, Phase III received ethical approval from China CDC (No. 201626). All findings are reported at disaggregated levels.

## Results

### Access to quality diagnosis

#### Diagnosis for smear-negative TB patients

Access to quality diagnosis for SS– TB improved substantially over the evaluation period. The percentage of the SS–patients who have further received either culture or RMT or both tests increased from 34.7% in 2015 to 87.4% in 2018. All three provinces have significantly improved diagnosis services during the study period. The percentage of SS– TB patients who received both sputum culture and RMT has increased from 0 to 34.3%. RMT was not widely used in 2015 in the study sites and no records were available. By 2018, over half (50.4%) of patients received RMT (Table 2).

**Table 2:** Percentage of culture and rapid molecular tests among smear-negative tuberculosis patients in 2015 and 2018

	2015					2018				
	No. of SS–patients	Receiving additional test				No. of SS–patients	Receiving additional test			
		Culture (%)	RMT (%)	RMT & Culture (%)	Total (%)		Culture (%)	RMT (%)	RMT and culture (%)	Total (%)
Zhejiang	536	158 (29.5)	0	0	158 (29.5)	472	265 (56.1)	42 (8.9)	84 (17.8)	391 (82.8)
Jilin	948	306 (32.3)	0	0	306 (32.3)	867	303 (34.9)	90 (10.4)	359 (41.4)	752 (86.7)
Ningxia	429	199 (46.4)	0	0	199 (46.4)	289	35 (12.1)	129 (44.6)	116 (40.1)	280 (96.9)
Total	1913	663 (34.7)	0	0	663 (34.7)	1628	603 (37.0)	261 (16.0)	559 (34.3)	1623 (87.4)

Source: TB Information Management System. *SS–* sputum smear negative, *RMT* rapid molecular test

#### Diagnosis for bacteriologically confirmed TB patients

**Fig. 1:**
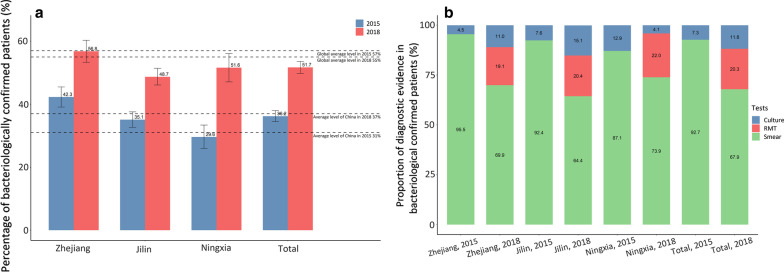
Percentage of bacteriological-confirmed TB patients (**a**) and distribution of diagnostic tests (**b**) in 2015 and 2018, disaggregated by province. Source: TB Information Management System and Global Tuberculosis Report 2016 and 2019. *TB* tuberculosis, *RMT* rapid molecular tests

The proportion of bacteriologically confirmed TB cases increased substantially during the study period and the RMT has been widely used in 2018. The percentage of bacteriological confirmed TB cases increased from 36.2% in 2015 to 51.7% in 2018 (Fig. 1) with a growth rate of 15.5% (*P* < 0.05). Compared to the national average, two provinces (Zhejiang and Jilin) performed above the national average (31%) in 2015. By 2018, all three provinces had a much higher proportion of bacteriologically confirmed TB cases than the national average level. Ningxia made the most progress, with 22.0% increase, followed by Jilin (16.3%), and Zhejiang (14.5%). Zhejiang had the highest proportion of pulmonary TB cases confirmed by bacteriology (42.3%) amongst the three provinces while in 2018, all three provinces had nearly 50% or even higher proportion of pulmonary TB cases confirmed by bacteriology.

The use of different diagnostic tests in identifying bacteriologically confirmed TB cases has shifted over time (Fig. 1). In 2015, over 92% of bacteriologically confirmed TB cases were diagnosed through sputum smear test and the remaining 7.3% were confirmed by sputum culture. In 2018, the RMT was used to diagnose 20.3% of bacteriologically confirmed TB cases. There was an increased share of TB cases confirmed by sputum culture in 2018 while less than 68% of patients were diagnosed by a sputum smear test. Ningxia had the highest proportion of bacteriologically confirmed TB cases diagnosed by sputum culture in 2015 (12.9%). By 2018, this proportion dropped to only 4.1%. In 2018, 22% of bacteriologically confirmed TB patients in Ningxia were diagnosed by RMT, followed by nearly 20.4% in Jilin and 19.1% in Zhejiang.

#### Diagnosis for drug susceptibility

**Fig. 2:**
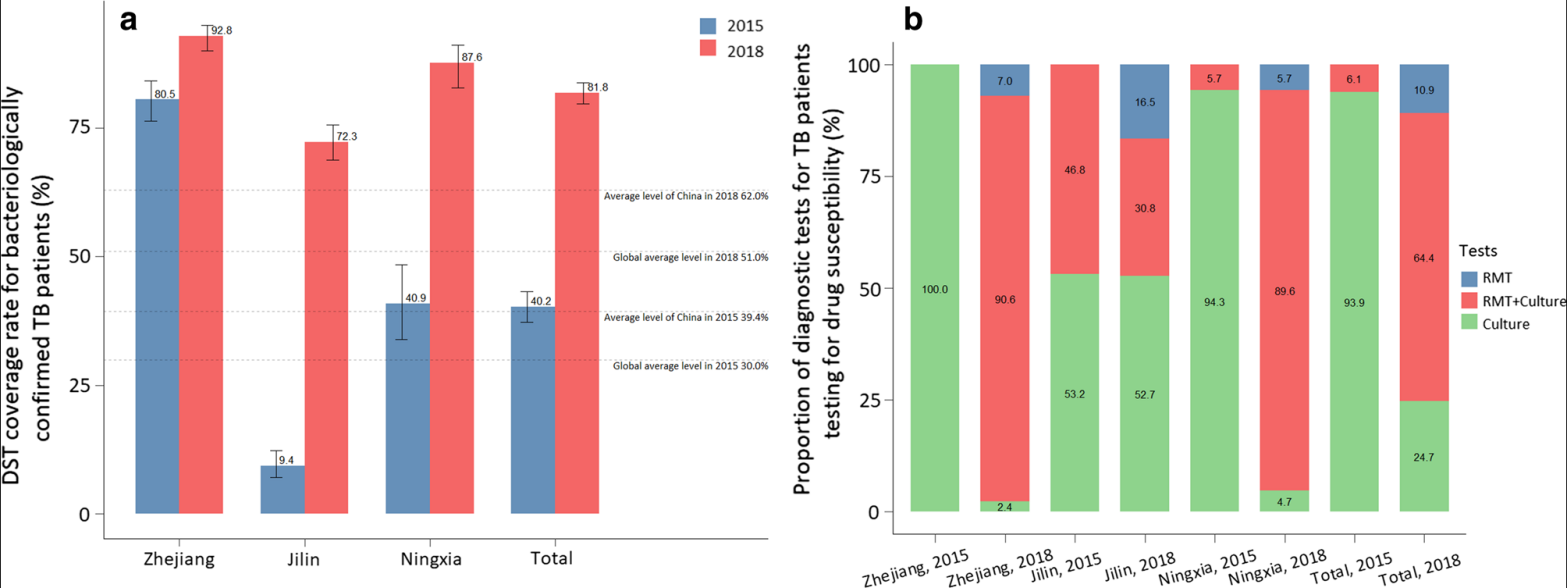
DST coverage rate (**a**) and distribution of different drug susceptibility tests method (**b**) for bacteriologically confirmed TB patients in 2015 and 2018, disaggregated by province. Source: TB Information Management System and Global Tuberculosis Report 2016 and 2019. *DST* drug susceptibility test, *TB* tuberculosis, *RMT* rapid molecular tests

As shown in Fig. 2, the proportion of TB patients with bacteriological confirmation who received DST doubled from 40.2% in 2015 to 81.8% in 2018. Zhejiang province maintained a relatively high proportion of DST (over 80%) during the study period, while both Jilin and Ningxia increased DST substantially, from 9.4% to 72.3% and 40.9% to 87.6%, respectively. In 2015, the proportion of performing DST in Ningxia was slightly higher than the national average level, while this proportion in Jilin was much lower than the national average level. At the end of the study period, the proportions of performing DST in all three provinces were higher than the national average. In addition, only 6.1% of patients received RMT in 2015, while this proportion increased to over 75% in 2018 (Fig. 2).

#### Adequate diagnostic services among TB patients

**Fig. 3:**
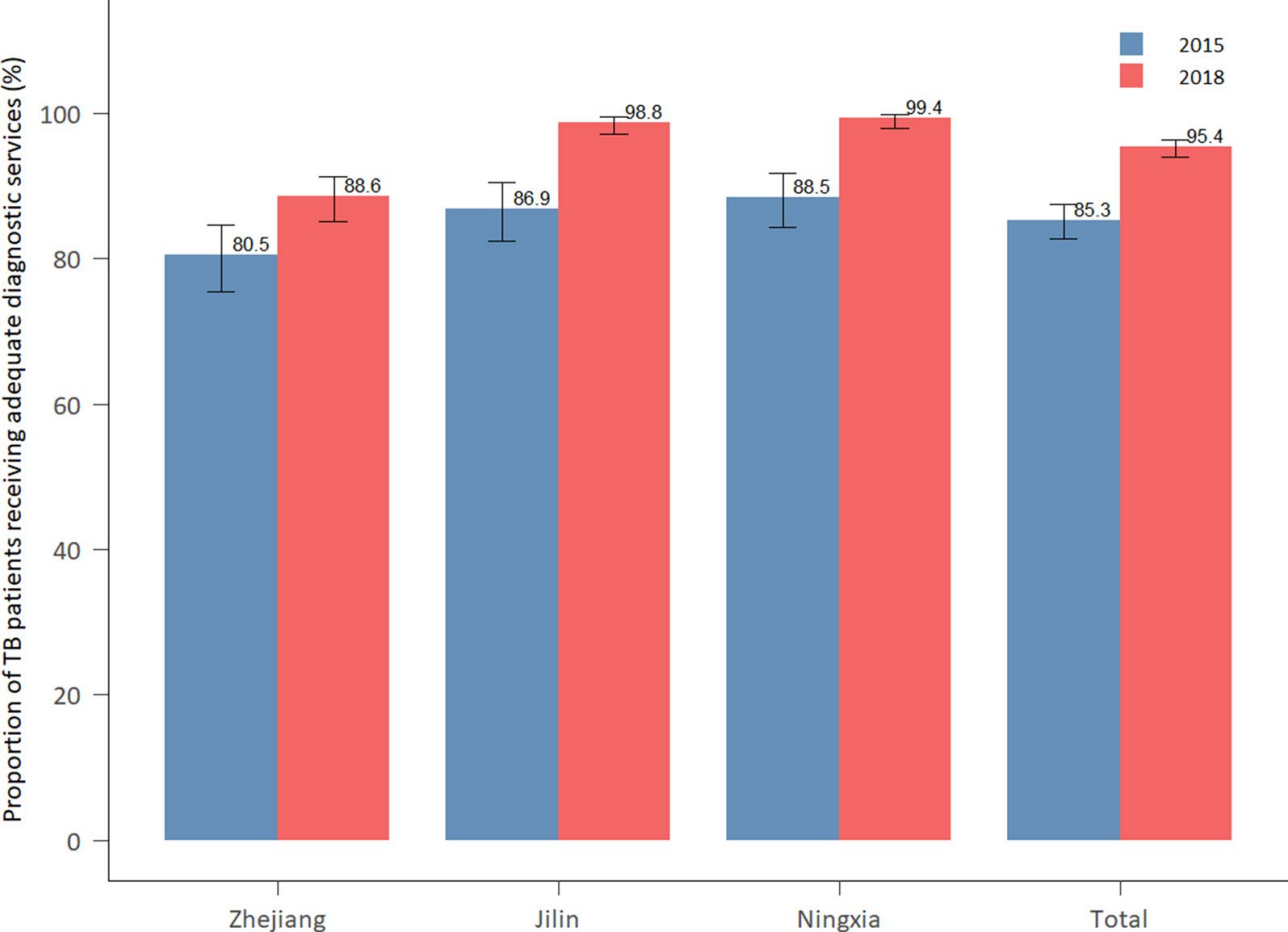
Proportion of tuberculosis patients received adequate diagnostic services in 2015 and 2018, disaggregated by province. Source: medical record review. *TB* Tuberculosis

The proportion of TB patients receiving adequate diagnostic services increased from 85.3 to 95.4% during the study period (Fig. 3). In the stratification of the analysis by province, this proportion increased in all three provinces by at least 8% during the study period. Of which, Ningxia had the highest proportion of TB patients receiving adequate diagnostic services.

**Fig. 4:**
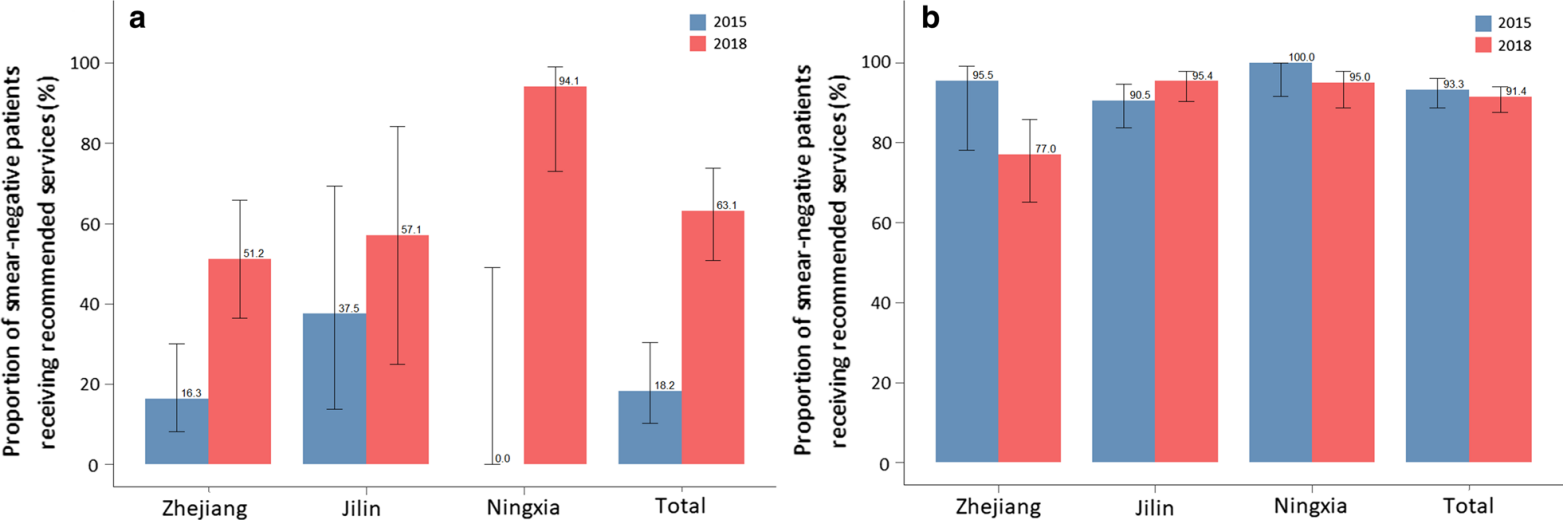
Proportion of smear-negative TB patients received recommended diagnostic services by Guideline in 2015 and 2018, disaggregated by province. **a** Among asymptomatic pulmonary TB patients, **b** among symptomatic pulmonary TB patients. Source: medical record review. *TB* tuberculosis

In 2015, 75% of all TB patients without bacteriological confirmation received the recommended diagnostic services outlined in the guideline. By 2018, this share was over 86%. Among asymptomatic TB patients (24.3% in 2015 and 18.3% in 2018), the proportion of SS– TB patients that received the recommended diagnostic services increased substantially from 18.2% in 2015 to 63.1% in 2018 (Fig. 4). In comparison, among TB patients with symptoms (76.7% in 2015 and 81.7% in 2018), the overall proportion of SS– TB patients that received the recommended diagnostic services declined slightly. The proportion of different tests received by TB patients is presented in Additional file 1: Appendix 3.

### Rational prescriptions of TB clinical services

#### Proportion of TB patients treated with irrational second-line drugs (SLDs)

**Fig. 5:**
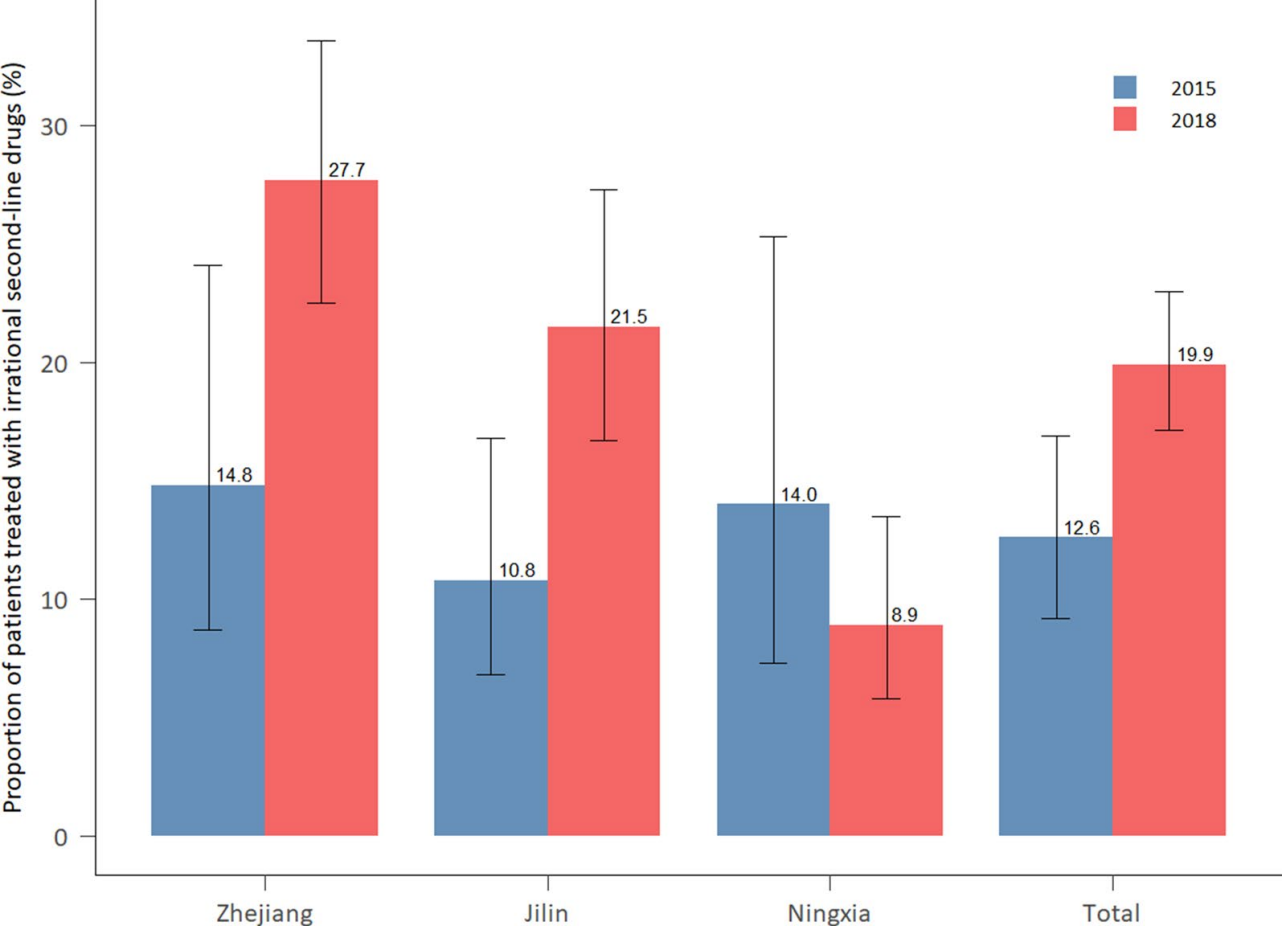
Proportion of drug susceptible TB patients treated with irrational second-line drug in 2015 and 2018, disaggregated by province. Source: medical record review. *TB* tuberculosis

The proportion of TB patients treated with irrational SLDs increased from 12.6% in 2015 to 19.9% in 2018 (Fig. 5). Over 27.7% of TB patients in Zhejiang received irrational SLDs in 2018, followed by Jilin and Ningxia. Zhejiang also witnessed the most increase in the proportion of TB patients treated by irrational SLDs between 2015 and 2018, followed by Jilin.

#### Proportion of TB patients receiving recommended follow-up examinations during the treatment course

**Fig. 6:**
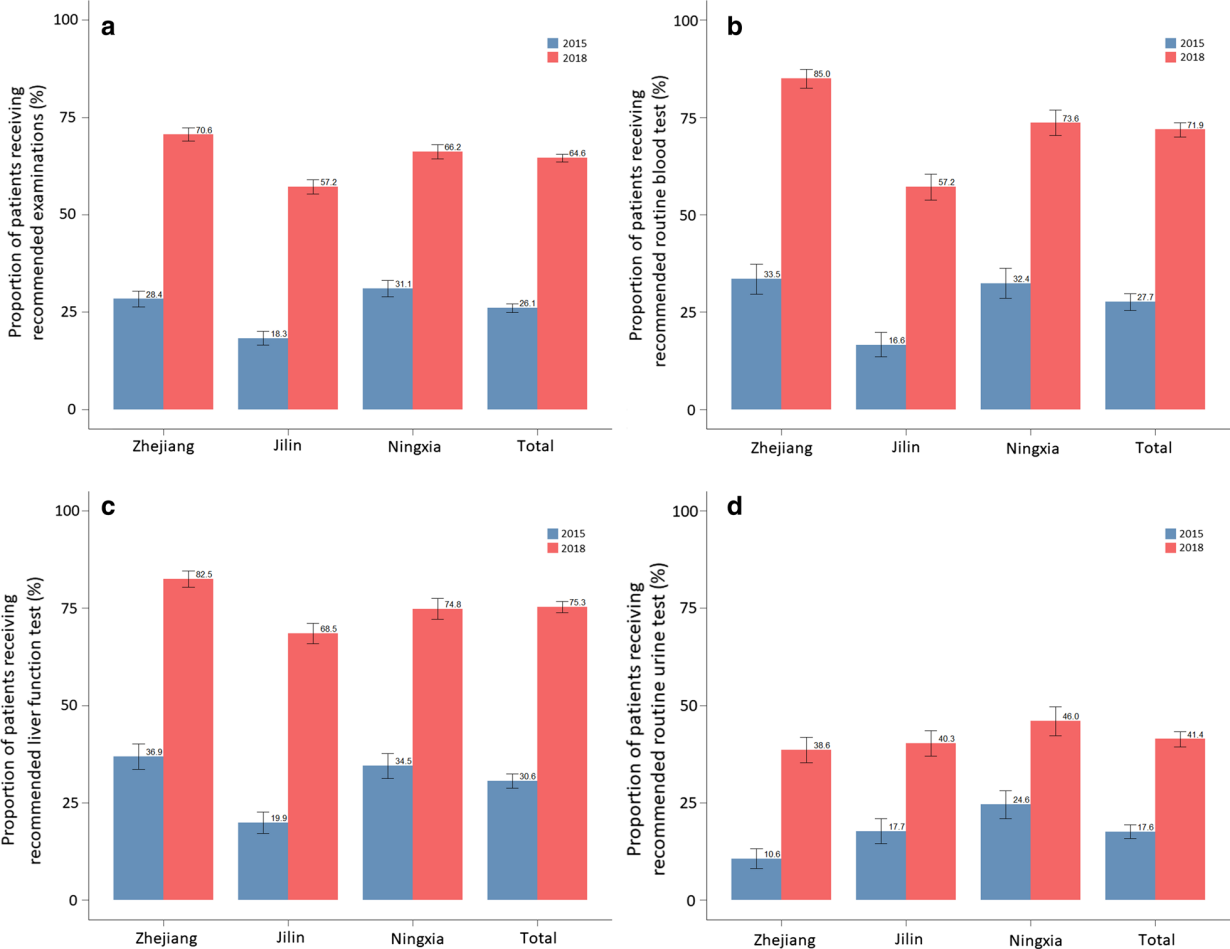
Proportion of TB patients receiving recommended follow-up examinations during treatment course in 2015 and 2018, disaggregated by province. **a** All test, **b** routine blood test, **c** liver function test, **d** routine urine test. Source: medical record review. *TB* tuberculosis

Figure 6 shows the changes in the proportion of TB patients receiving the recommended follow-up examinations during the treatment course. The proportion of patients receiving all recommended tests increased from 26.1% in 2015 to 64.6% in 2018. All three provinces have made substantial progress. In 2015, the proportion of patients received all recommended follow-up examination was the highest in Ningxia, followed by Zhejiang and Jilin. In 2018, this proportion was the highest in Zhejiang, followed by Ningxia, and the lowest in Jilin. Given different types of tests, the proportion of patients who received proper routine blood tests increased the most, from 27.7% in 2015 to 71.9% in 2018. The proportions of patients who received proper routine blood tests and liver function tests were higher in Zhejiang, compared to the other two provinces. A higher proportion of patients received routine urine tests in Ningxia than that in Zhejiang and Jilin. The proportion of patients that received the minimal recommended tests can be found in Additional file 1: Appendix 3. We also present the proportion of different tests received by TB patients in Additional file 1: Appendix 4.

## Discussion

Our study found that between 2015 and 2018, access to quality diagnosis for TB improved substantially in the study sites, mainly attributed to the increased use of RMT and improved diagnostic services for SS– patients. However, access to rational treatment had mixed findings and regional disparities remained, but the gaps across the study sites were narrowed for almost all indicators.

We observed significant improvements in access to quality TB diagnosis measuring by various indicators after the implementation of the China-Gates TB project Phase III. The introduction of RMT is one of the major contributors to improvement in access to quality TB diagnosis. Similar to other studies, our study proved that the introduction of RMT increased the diagnosis of DR-TB [18–20]. The impact of the China-Gates TB project Phase III was beyond the application of RMT. The capacity training activities have improved the access to quality diagnosis of bacteriologically confirmed TB patients and TB patients without bacteriological confirmation as well. The improved diagnosis is critical for achieving desired treatment results. The China-Gates TB project has improved the performance of the overall TB care system in the project sites through comprehensive interventions with the strong commitment of the local governments. For instance, comparing to non-project sites, the project sites issued more supporting policies for TB including financial support for TB and drug-resistant TB patients, etc.

China introduced DOTs in the 1990s, which effectively improved the completion rate of TB treatment [21]. However, the detection of TB and DR-TB remains a barrier for furtherer improvement to TB control in China [22]. Compared to the global average of 57% in 2015, the proportion of TB cases confirmed by bacteriology in China was only 31% [23]. The majority of TB cases in China are diagnosed through symptoms, chest radiography, pathology, or histology, which have low specificity and could potentially delay treatment for TB [24]. Parsons et al. reported that the specificity of sputum smear was largely determined by the quality of sputum sample, capacity and experiences of the technicians and the device [25]. Without a sensitive and timely diagnosis, the treatment for TB could potentially be delayed and lead to further spread of TB [26].

New TB diagnostic devices haven’t been widely introduced in China for many reasons. There is no domestic funding to support the implementation of GeneXpert in most regions of China. In addition, the number of staff trained to perform TB diagnosis is insufficient [27]. The RMT was scaled up through the China–Gates TB Project Phase III in the three project provinces, which have contributed to the improvement of detecting TB patients. The training activities also improved the capacity of staff involving in the TB diagnosis. The proportion of bacteriologically confirmed TB cases in the three project provinces (36.2%) was slightly above the national average (31%) in 2015, while in 2018, the proportion of bacteriologically confirmed TB cases in the three provinces (52%) has almost reached the global average (55%). The application of RMT essentially improved the diagnosis of TB, which further promoted the treatment and control of TB. We also observed the regional gaps in access to quality diagnosis have been narrowed. DST coverage for bacteriologically confirmed TB patients doubled from 40.2% in 2015 to 81.8% in 2018. More patients who were not belong to the five high-risk groups, including chronic TB cases, close contacts of MDR-TB patients, patients with treatment failure, relapsed and returned patients, and smear-positive patients at the end of the third month of initial treatment, had access to the drug susceptibility tests in project areas. Yang et al. reported that the screening among high-risk groups of drug-resistant TB would miss the detection of more than half of the MDR-TB patients, while the application of RMT and expanding the targets of drug resistance screening improved the detection rate of RR-TB patients [28].

Infectious disease control is a public good and should be a top priority for the government. Our findings indicate that improved quality and reduced regional disparity of TB diagnosis by using RMT and it is necessary to develop a sustainable domestic financing mechanism to further scale up the RMT across the country.

Unfortunately, access to rational treatment hasn’t made substantial progress. Irrational use of anti-TB drugs has been a long-lasting issue in China for many reasons [29]. The mark-up from medicines has provided adverse financial incentives for providers to prescribe expensive drugs. The situation is even worse for TB. Under the current TB control model in China, TB services are provided at TB-designated hospitals in most regions. Essential first-line TB drugs, smear tests and chest X-rays are fully subsidized by the central government and are provided for free. To obtain financial profits, the prescription of second-line drugs has become common. Meanwhile, the wide coverage of health insurance schemes in China has become an incentive for patients and healthcare providers to pursuing more medical services, including auxiliary medications and follow-up tests.

Over-prescribing medical services could increase the financial burden to TB patients, even lead to some adverse health outcomes (e.g. drug resistance). Our findings on the increasing use of SLD call for more attention on the regulation of clinical treatment for TB. Financing policies, along with other support policies, should be implemented synergically to promote rational treatment for TB.

Our study attempted to evaluate the impacts of comprehensive TB control models with a wide range of interventions on access to quality diagnosis and rational treatment. We employed a set of indicators to track the changes of access to quality diagnosis and rational treatment, but our study still has some limitations. First, our observation was limited within the TB care system and the availability of data from TBIMS and medical records. No considerations have been given to other factors such as socioeconomic characters at the patient level or the overall health system differences, which affected the extrapolation of the results. Second, several study sites have a relatively small number of TB patients, which is another limitation of our study. Third, we employed a before and after study design without controlling all the potential factors that might affect the outcome indicators. Other health policies, and factors such as economic development, might also contribute to the improved results. Finally, the data from TBIMS is designed for routine administration. It does not necessarily have robust quality assurance, and errors in data entry cannot be ruled out.

## Conclusions

Our study found that the access to quality TB diagnosis and follow-up services has been improved substantially, however, irrational use of SLDs remains a problem in the study sites after the implementation of the China-Gates TB Project. The widely use of RMT and extending DST for bacteriologically confirmed TB patients could improve the access to quality TB diagnosis of TB.

## Supplementary Information

**Additional file 1.** **Appendix 1.** Basic information of study sites. **Appendix 2.** Checklist for medical record review. **Appendix 3.** Figure A3.1 Proportion of different tests used by TB patients A) among asymptomatic pulmonary TB patients B) among symptomatic pulmonary TB patients. Figure A3.2 Proportion of TB patients receiving minimal recommended follow-up examinations during treatment course in 2015 and 2018, disaggregated by province A) all test B) routine blood test C) liver function test D) routine urine test. **Appendix 4.** Table A4.1 Coverage of smear culture and RMT among smear-negative patients. Table A4.2 Coverage of different types of TB diagnostic tests among smear-negative patients. Table A4.3 Percentage of pulmonary TB cases confirmed by bacteriology. Table A4.4 Proportion of diagnostic tests in bacteriological-confirmed TB patients. Table A4.5 DST coverage for bacteriologically confirmed TB patients. Table A4.6 Proportion of diagnostic tests for bacteriologically confirmed TB patients testing for drug susceptibility. Table A4.7 Proportion of TB patients received adequate diagnostic services. Table A4.8 Proportion of smear-negative TB patients received recommended diagnostic services by Guideline. Table A4.9 Proportion of drug susceptible TB patients treated by second-line drug (SLD). Table A4.10 Proportion of TB patients receiving recommended follow-up examinations during treatment course in 2015 and 2018, disaggregated by province A) all test B) blood routine test C) liver function test D) liver function test (routine urine test)routine. Table A4.11 Proportion of TB patients receiving recommended follow-up examinations during treatment course in 2015 and 2018, disaggregated by province.

## Data Availability

The datasets generated and analysed during the current study are not publicly available due to the regulations of China CDC. Readers of the article need to discuss with China CDC and obtain their permission before the release of the dataset.

## Abbreviations

ADR: Adverse drug reactions
BMGF: Bill and Melinda Gates Foundation
China CDC: Chinese Center for Disease Control and Prevention
CT: Computed tomography
DALYs: Disability-adjusted life years
DOTS: Directly observe treatment strategy
DST: Drug susceptibility test
EMMs: Electronic medicine management system
MDR-TB: Multidrug-resistant tuberculosis
NHC: The Chinese National Health Commission
RMT: Rapid molecular tests
RR-TB: Rifampicin resistant tuberculosis
SLDs: Second-line drugs
SS: Sputum smear negative
TB: Tuberculosis
TBIMS: Tuberculosis Information Management System
WHO: World Health Organization
