# Supplementary material for: Access to quality diagnosis and rational treatment for tuberculosis: real-world evidence from China–Gates Tuberculosis Control Project Phase III
Source: Infect Dis Poverty. 2021 Jun 29;10:92. doi: 10.1186/s40249-021-00875-8 (PMC8243738; doi:10.1186/s40249-021-00875-8)
Supplement: Supplementary file 1 — Additional file 1. Appendix 1. Basic information of study sites. Appendix 2. Checklist for medical record review. Appendix 3. Figure A3.1 Proportion of different tests used by TB patients A) among asymptomatic pulmonary TB patients B) among symptomatic pulmonary TB patients. Figure A3.2 Proportion of TB patients receiving minimal recommended follow-up examinations during treatment course in 2015 and 2018, disaggregated by province A) all test B) routine blood test C) liver function test D) routine urine test. Appendix 4. Table A4.1 Coverage of smear culture and RMT among smear-negative patients. Table A4.2 Coverage of different types of TB diagnostic tests among smear-negative patients. Table A4.3 Percentage of pulmonary TB cases confirmed by bacteriology. Table A4.4 Proportion of diagnostic tests in bacteriological-confirmed TB patients. Table A4.5 DST coverage for bacteriologically confirmed TB patients. Table A4.6 Proportion of diagnostic tests for bacteriologically confirmed TB patients testing for drug susceptibility. Table A4.7 Proportion of TB patients received adequate diagnostic services. Table A4.8 Proportion of smear-negative TB patients received recommended diagnostic services by Guideline. Table A4.9 Proportion of drug susceptible TB patients treated by second-line drug (SLD). Table A4.10 Proportion of TB patients receiving recommended follow-up examinations during treatment course in 2015 and 2018, disaggregated by province A) all test B) blood routine test C) liver function test D) liver function test (routine urine test)routine. Table A4.11 Proportion of TB patients receiving recommended follow-up examinations during treatment course in 2015 and 2018, disaggregated by province. [file 40249_2021_875_MOESM1_ESM.docx]

# **APPENDIX**

## **Appendix 1 Basic information of study sites**

**Table A1 Socioeconomics and TB epidemiology in study sites, 2015**

|  | Population（million） | Proportion of agriculture population(%) | GDP per capita (CNY) | Notified TB incidence (per 100 000 population) |
| --- | --- | --- | --- | --- |
| **Zhejiang Province** | **55.39** | **34.20** | **77,644** | **49.4** |
| Jiashan | 0.39 | 58.60 | 109,811 | 38.7 |
| Tongxiang | 0.69 | 42.60 | 94,874 | 31.2 |
| Longyou | 0.37^2^ | 64.21^1^ | 27,240 | 63.5 |
| Changshan | 0.34 | 69.10 | 32,580 | 54.7 |
| **Jilin Province** | **27.53** | **44.70** | **51,851** | **45.7** |
| Nongan | 1.15 | 80.4^3^ | 35,261 | 42.3 |
| Dehui | 0.93 | 81.90 | 43,270 | 53.8 |
| Zhenlai | 0.27 | 58.60 | 49,440 | 90.1 |
| Taonan | 0.42 | 63.20 | 33,144 | 28.0 |
| **Ningxia Province** | **6.67** | **44.70** | **44,015** | **37.6** |
| Haiyuan | 0.46^2^ | 80.70^2^ | 10,716^2^ | 28.3 |
| Zhongning | 0.34 | 59.10 | 37,130 | 36.0 |
| Tongxin | 0.38^2^ | 71.3^2^ | 14,528^2^ | 37.2 |
| Qingtongxia | 0.28 | 41.70 | 45,575 | 26.2 |

Notes: 1CNY = 0.16USD in 2015

Due to lack of data for 2015, we used the data from nearest year available. 1: in 2010; 2: in 2016; 3: in 2017.

## **Appendix 2 Checklist for medical record review**

**______Province_______Prefecture________County_________Name of hospital**

| Patient code | | | |  | | | Sex | | M□ F□ | | | | | | | Age | | | | |  | | | #Medical record | | | |  | | | | | Treatment type | | | New□ Relapse□ | | | |  |
| --- | --- | --- | --- | --- | --- | --- | --- | --- | --- | --- | --- | --- | --- | --- | --- | --- | --- | --- | --- | --- | --- | --- | --- | --- | --- | --- | --- | --- | --- | --- | --- | --- | --- | --- | --- | --- | --- | --- | --- | --- |
| Date of diagnosis | | | |  | | | Type of patient | | TB□ DR-TB□ | | | | | | | Type of DR | | | | | H□ R□ MDR□ Injection resistant MDR□ Quinolone resistance□ Extensive resistance □ | | | | | | | | | | | | | | | | | | |  |
| **Diagnosis** | | | | | | | | | | | | | | | | | | | | | | | | | | | | Adequate diagnosis | | | | | | | | Yes□ No□ | | | |  |
| Number of sputum smears submitted for examination | | | | 0□ 1□ 2□ 3□ | | | | | | | | | | | | Smear result | | | | | Negative□ Positive □ | | | | | | | | | | | | | | | | | | |  |
| Phenotypic sputum culture | | | | No□ Negative□ Positive□ | | | | | | | | | | | | Cultivation method | | | | | L-J □ M960/460 □ Micropore-plate method(Microdrug sensitivity, MicroDST □ | | | | | | | | | | | | | | | | | | |  |
| Phenotypic susceptibility results | | | | No□ Susceptible □ Drug resistance□ | | | | | | | | | | | | Drug resistance | | | | | Isoniazid □ Rifampicin□ ofloxacin (Ofx) □ levofloxacin (Lfx) □ moxifloxacin (Mfx) □ KM□ AK□ CM□ SM□ | | | | | | | | | | | | | | | | | | |  |
| Molecular diagnosis of drug resistance | | | | No□ Susceptible □ Drug resistance□ | | | | | | | | | | | | Drug resistance | | | | | H□ R□ E□ SM□ Quinolone□ | | | | | | | Detection method | | | | | Hain□ Xpert□ Gene chip □  Fusion curve □ Other □ please describe: ___________ | | | | | | |  |
| X ray | | | | No□ Yes□ | | | | | | | | | | | | CT | | | | | No□ Yes□ | | | | | | | Contact history of TB patients | | | | | No□ Yes□ | | | | | | |  |
| PPD | | | | No□ Negative□ Positive□ | | | | | | | | | | | | Anti-tuberculosis antibody | | | | | No□ Negative□ Positive□ | | | | | | | IGRAs | | | | | No□ Negative□ Positive□ | | | | | | |  |
| TB symptoms | | | | No□ Cough□ Expectoration □ Fever□ Hemoptysis □ | | | | | | | | | | | | Anti-inflammatory treatment | | | | | No□ Rational anti-inflammatory□ Irrational anti-inflammatory (No assessment, use of fluoroquinolone or aminoglycoside anti-inflammatory) □ | | | | | | | | | | | | | | | | | | |  |
| Tumor marker | | | | No□ Normal□ Abnormal □，CEA_________ ug/ml，other abnormal, please describe_________ | | | | | | | | | | | | | | | | | | | | | | | | Exfoliated cells | | | | | No□ Negative□ Positive□ | | | | | | |  |
| Bronchoscopy | | | | Normal□ Abnormal □ please describe: ________ | | | | | | | | | | | | Lung or other biopsy | | | | | No□ Normal□ Abnormal□ please describe: ___________ | | | | | | | | | | | | | | | | | | |  |
| **Reasons for hospital admission (choose all that apply)** | | | | | | | | | | | | | | | | | | | | | | | | | | | | | | | | | | | | | | | |  |
| Reasons for hospital admission | | | | TB：1TB□ 2 Hematogenous disseminated TB □ 3 Tubercular meningitis□ 4 Pleurisy □ 5 Peritonitis □ 6 Pericarditis □ 7Surgery for TB□ 8 Body temperature≥38.5℃□ | | | | | | | | | | | | | | | | | | | | | | | | | | | | | | | | | | | |  |
|  |  |  |  | Differential diagnosis：9 Anti-inflammatory □ 10Tumor □ 11 Biopsy □ | | | | | | | | | | | | | | | | | | | | | | | | | | | | | | | | | | | |  |
|  |  |  |  | TB complications：12 Hemoptysis □ 13 Pneumothorax □ 14 Respiratory failure□ 15 Intestinal obstruction □ | | | | | | | | | | | | | | | | | | | | | | | | | | | | | | | | | | | |  |
|  |  |  |  | Adverse reactions：16Allergic□ 17Liver damage□ 18Renal damage□ 19 Bone marrow suppression □ 20 Electrolyte disturbance □ 21 Gastrointestinal symptoms □ | | | | | | | | | | | | | | | | | | | | | | | | | | | | | | | | | | | |  |
|  |  |  |  | Comorbidities：22 Diabetes□ 23 Coronary Heart Disease □ 24 Cerebrovascular Disease □ 25Infection, body temperature ≥38.5℃□ 26Insurance policy□ 27Other, please describe____ | | | | | | | | | | | | | | | | | | | | | | | | | | | | | | | | | | | |  |
| **Treatment plan and outcome** | | | | | | | | | | | | | | | | | | | | | | | | | | | | | | | | | | | | | | | |  |
| Treatment plan | | Include treatment | | | | | No□ Yes□ | | | | Initial standard chemotherapy regimen | | | | | | | | No□ Yes□ | | | | | Adjustment | | | No□ Yes□ | | | | | Date of starting treatment | | | | |  | | |  |
|  |  | Is the adjustment reasonable | | | | | No□ Yes□ | | | | Reason for adjustment | | | 1 Adverse reaction□ 2Financial burden□ 3 Shortage of medicine □ 4 No injection conditions □ 5Severe TB□ 6 Complications □ 7Aged 70 years or older □ 8Other, please describe______ | | | | | | | | | | | | | | | | | | | | | | | | | |  |
| Treatment outcome assessment for TB | | At the end of second months for initial treatment / third months for retreatment | | | | | | | | | Sputum | | | No□ Negative□ Positive□ | | | | | | | | | | Image | | | No□ Improved□ Same□ Deterioration □ | | | | | | | | | | | | |  |
|  |  | At the end of sixth months for initial treatment / eighth months for retreatment | | | | | | | | | Sputum | | | No□ Negative□ Positive□ | | | | | | | | | | Image | | | No□ Improved□ Same□ Deterioration □ | | | | | | | | | | | | |  |
|  |  | Treatment outcome | | | | | 1Cure□ 2Finish treatment□ 3TB death□ 4Non-TB death□ 5Treatment failure□ 6lose follow-up□ 7Adverse reaction and pause treatment□ 8Change diagnosis□ 9Refuse treatment □ 10Become MDR-TB□ 11Not able to assess□ | | | | | | | | | | | | | | | | | | | | | | | | | | | | | | | | |  |
| Treatment outcome assessment for DR-TB | | At the end of second months for initial treatment / third months for retreatment | | | | | | | | | Sputum | | | No□ Negative□ Positive□ | | | | | | | | | | Image | | | No□ Improved□ Same□ Deterioration □ | | | | | | | | | | | | |  |
|  |  | At the end of sixth months for initial treatment / eighth months for retreatment | | | | | | | | | Sputum | | | No□ Negative□ Positive□ | | | | | | | | | | Image | | | No□ Improved□ Same□ Deterioration □ | | | | | | | | | | | | |  |
|  |  | Treatment outcome | | | | | 1Cure□ 2Finish treatment□ 3TB death□ 4Non-TB death□ 5Treatment failure□ 6lose follow-up□ 7Adverse reaction and pause treatment□ 8Change diagnosis□ 9Refuse treatment □ 10Become MDR-TB□ 11Not able to assess□ | | | | | | | | | | | | | | | | | | | | | | | | | | | | | | | | |  |
| **Diagnostic tests (by months)** | | | | | | | | | | | | | | | | | | | | | | | | | | | | | | | | | | | | | | | |  |
| Test | 0 | | 1 | | 2 | 3 | 4 | 5 | | 6 | | 7 | 8 | | 9 | | 10 | 11 | | 12 | | 13 | 14 | | 15 | 16 | | | 17 | 18 | 19 | | | 20 | 21 | | 22 | 23 | 24 | |
| Blood routine |  | |  | |  |  |  |  | |  | |  |  | |  | |  |  | |  | |  |  | |  |  | | |  |  |  | | |  |  | |  |  |  | |
| Liver function |  | |  | |  |  |  |  | |  | |  |  | |  | |  |  | |  | |  |  | |  |  | | |  |  |  | | |  |  | |  |  |  | |
| Renal function |  | |  | |  |  |  |  | |  | |  |  | |  | |  |  | |  | |  |  | |  |  | | |  |  |  | | |  |  | |  |  |  | |
| Urine routine |  | |  | |  |  |  |  | |  | |  |  | |  | |  |  | |  | |  |  | |  |  | | |  |  |  | | |  |  | |  |  |  | |
| Electrolyte |  | |  | |  |  |  |  | |  | |  |  | |  | |  |  | |  | |  |  | |  |  | | |  |  |  | | |  |  | |  |  |  | |
| TSH |  | |  | |  |  |  |  | |  | |  |  | |  | |  |  | |  | |  |  | |  |  | | |  |  |  | | |  |  | |  |  |  | |
| Hearing |  | |  | |  |  |  |  | |  | |  |  | |  | |  |  | |  | |  |  | |  |  | | |  |  |  | | |  |  | |  |  |  | |
| Vision |  | |  | |  |  |  |  | |  | |  |  | |  | |  |  | |  | |  |  | |  |  | | |  |  |  | | |  |  | |  |  |  | |
| Sputum smear |  | |  | |  |  |  |  | |  | |  |  | |  | |  |  | |  | |  |  | |  |  | | |  |  |  | | |  |  | |  |  |  | |
| Sputum Culture |  | |  | |  |  |  |  | |  | |  |  | |  | |  |  | |  | |  |  | |  |  | | |  |  |  | | |  |  | |  |  |  | |
| X Ray |  | |  | |  |  |  |  | |  | |  |  | |  | |  |  | |  | |  |  | |  |  | | |  |  |  | | |  |  | |  |  |  | |
| CT |  | |  | |  |  |  |  | |  | |  |  | |  | |  |  | |  | |  |  | |  |  | | |  |  |  | | |  |  | |  |  |  | |
| Weight |  | |  | |  |  |  |  | |  | |  |  | |  | |  |  | |  | |  |  | |  |  | | |  |  |  | | |  |  | |  |  |  | |
| Take medicine |  | |  | |  |  |  |  | |  | |  |  | |  | |  |  | |  | |  |  | |  |  | | |  |  |  | | |  |  | |  |  |  | |

## **Appendix 3 Additional figures**


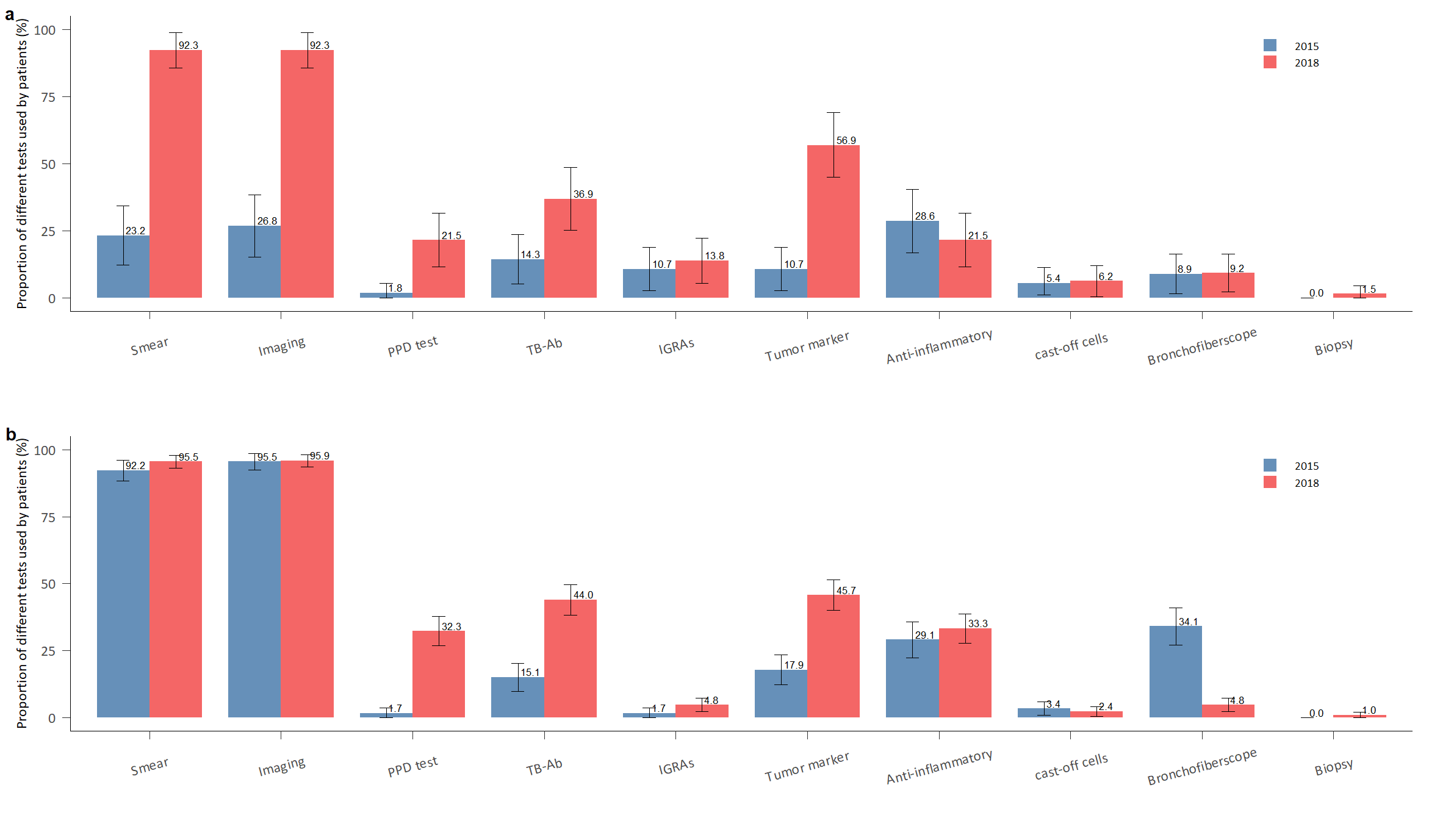


**Figure A3.1 Proportion of different tests used by TB patients A) among asymptomatic pulmonary TB patients B) among symptomatic pulmonary TB patients. Abbreviations: TB- Tuberculosis. (Source: medical record review)**


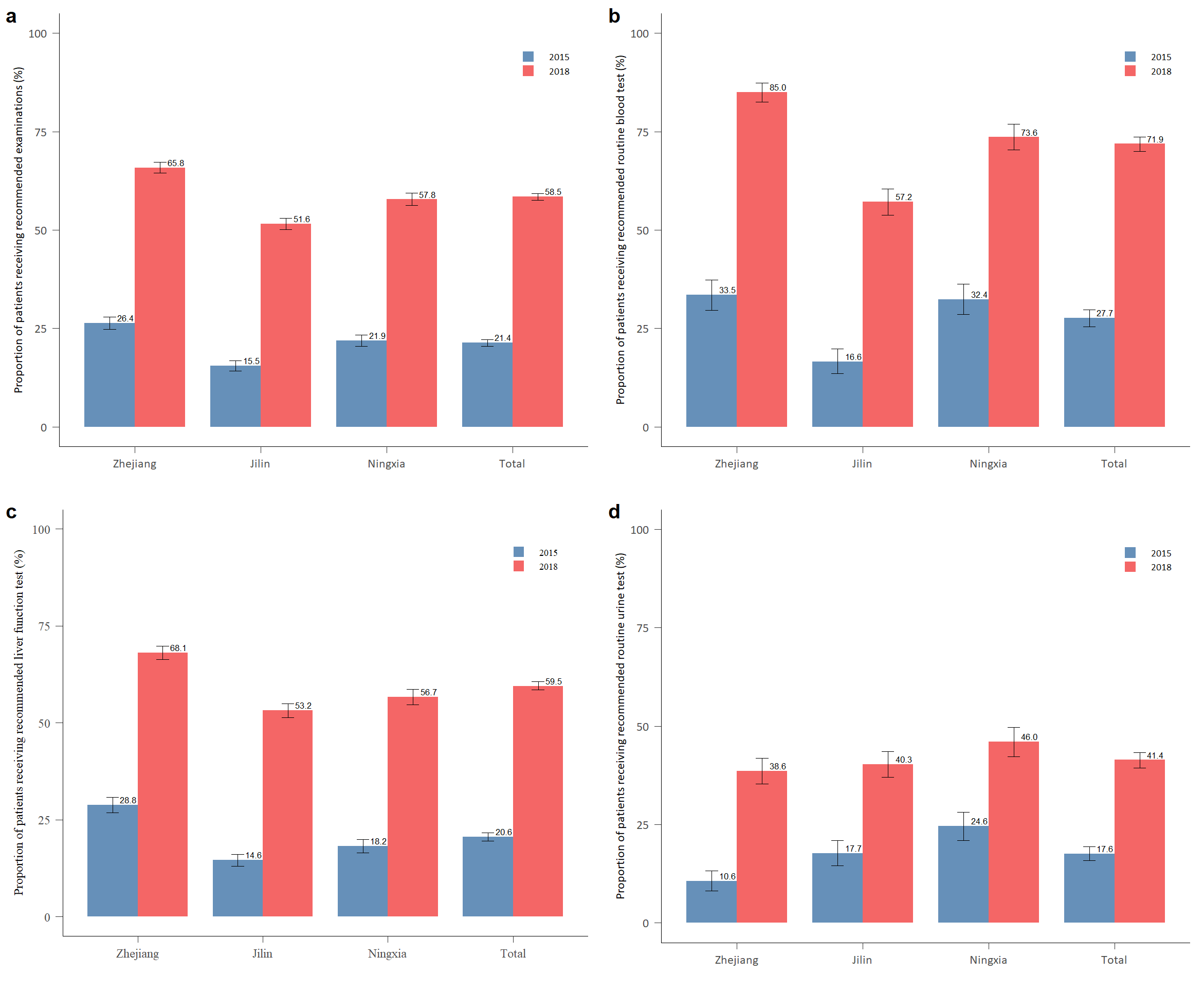


**Figure A3.2 Proportion of TB patients receiving minimal recommended follow-up examinations during treatment course in 2015 and 2018, disaggregated by province A) all test B) routine blood test C) liver function test D) routine urine test. Abbreviations: TB- Tuberculosis. (Source: medical record review)**

## **Appendix 4 Tables**

**Table A4.1 Coverage of smear culture and RMT among smear-negative patients**

| **Study sites** | **2015** | | |  | **2018** | | | **P value** |
| --- | --- | --- | --- | --- | --- | --- | --- | --- |
|  | **# Patients** | **# of tests *** | **Proportion (%)** |  | **# Patients** | **# of tests *** | **Proportion (%)** |  |
| Zhejiang | 536 | 158 | 29.5 |  | 472 | 391 | 82.8 | <0.001 |
| Jilin | 948 | 306 | 32.3 |  | 867 | 752 | 86.7 | <0.001 |
| Ningxia | 429 | 199 | 46.4 |  | 289 | 280 | 96.9 | <0.001 |
| **Total** | **1913** | **663** | **34.7** |  | **1628** | **1423** | **87.4** | **<0.001** |

Notes: total of smear culture and RMT

**Table A4.2 Coverage of different types of TB diagnostic tests among smear-negative patients**

| **Study sites** | **2015** | | | | |  | **2018** | | | | | | **P value** |
| --- | --- | --- | --- | --- | --- | --- | --- | --- | --- | --- | --- | --- | --- |
|  | **#Patients** | **#Smear (%)** | **#Smear**  **+ RMT (%)** | **#Smear +culture (%)** | **#Smear**  **+culture**  **+RMT (%)** |  | | **#Patients** | **#Smear (%)** | **#Smear**  **+ RMT (%)** | **#Smear +culture (%)** | **#Smear**  **+culture**  **+RMT (%)** |  |
| Zhejiang | 536 | 378 (70.5) | 0 | 158  (29.5) | 0 |  | | 472 | 81  (17.2) | 42  (8.9) | 265  (56.1) | 84  (17.8) | <0.001 |
| Jilin | 948 | 642  (67.7) | 0 | 306  (32.3) | 0 |  | | 867 | 115  (13.3) | 90  (10.4) | 303  (34.9) | 359  (41.4) | <0.001 |
| Ningxia | 429 | 230  (53.6) | 0 | 199  (46.4) | 0 |  | | 289 | 9  (3.1) | 129  (44.6) | 35  (12.1) | 116  (40.1) | <0.001 |
| **Total** | **1913** | **1250**  **(65.3)** | **0** | **663**  **(34.7)** | **0** |  | | **1628** | **205**  (12.6) | **261**  (16.0) | **603**  (37.0) | **559**  (34.3) | **<0.001** |

**Table A4.3 Percentage of pulmonary TB cases confirmed by bacteriology**

| **Study sites** | **2015** | | |  | **2018** | | | **P value** |
| --- | --- | --- | --- | --- | --- | --- | --- | --- |
|  | **#Patients** | **#Bacteriologically positive** | **Positive rate (%)** |  | **#Patients** | **#Bacteriologically positive** | **Positive rate (%)** |  |
| Zhejiang | 899 | 380 | 42.3 |  | 783 | 445 | 56.8 | <0.001 |
| Jilin | 1427 | 501 | 35.1 |  | 1356 | 661 | 48.7 | <0.001 |
| Ningxia | 578 | 171 | 29.6 |  | 467 | 241 | 51.6 | <0.001 |
| **Total** | **2904** | **1052** | **36.2** |  | **2606** | **1347** | **51.7** | **<0.001** |

**Table A4.4 Proportion of diagnostic tests in bacteriological-confirmed TB patients**

| **Study sites** | **2015** | | | |  | **2018** | | | | **P value** |
| --- | --- | --- | --- | --- | --- | --- | --- | --- | --- | --- |
|  | **#Patients** | **#Smear positive (%)** | **#RMT positive (%)** | **#Culture positive (%)** |  | **#Patients** | **#Smear positive (%)** | **#RMT positive (%)** | **#Culture positive (%)** |  |
| Zhejiang | 380 | 363(95.5) | 0 | 17(4.5) |  | 445 | 311(69.9) | 85(19.1) | 49(11.0) | <0.001 |
| Jilin | 501 | 463(92.4) | 0 | 38(7.6) |  | 661 | 426(64.4) | 135(20.4) | 100(15.1) | <0.001 |
| Ningxia | 171 | 149(87.1) | 0 | 22(12.9) |  | 241 | 178(73.9) | 53(22.0) | 10(4.1) | <0.001 |
| **Total** | 1052 | 975(92.7) | 0 | 77(7.3) |  | 1347 | 915(67.9) | 273(20.3) | 159(11.8) | <0.001 |

**Table A4.5 DST coverage for bacteriologically confirmed TB patients**

| **Study sites** | **2015** | | |  | **2018** | | | **P value** |
| --- | --- | --- | --- | --- | --- | --- | --- | --- |
|  | **#Patients** | **#DST tests** | **DST coverage (%)** |  | **#Patients** | **#DST tests** | **DST coverage (%)** |  |
| Zhejiang | 380 | 306 | 80.5 |  | 445 | 413 | 92.8 | <0.001 |
| Jilin | 501 | 47 | 9.4 |  | 661 | 478 | 72.3 | <0.001 |
| Ningxia | 171 | 70 | 40.9 |  | 241 | 211 | 87.6 | <0.001 |
| **Total** | **1052** | **423** | **40.2** |  | **1347** | **1102** | **81.8** | **<0.001** |

**Table A4.6 Proportion of diagnostic tests for bacteriologically confirmed TB patients testing for drug susceptibility**

| **Study sites** | **2015** | | | |  | **2018** | | | | **P value** |
| --- | --- | --- | --- | --- | --- | --- | --- | --- | --- | --- |
|  | **#Patients** | **#Culture (%)** | **#RMT+culture (%)** | **#RMT (%)** |  | **#Patients** | **#Culture (%)** | **#RMT+culture (%)** | **#RMT (%)** |  |
| Zhejiang | 306 | 306(100.0) | 0(0.0) | 0(0.0) |  | 413 | 10(2.4) | 374(90.6) | 29(7.0) | <0.001 |
| Jilin | 47 | 25(53.2) | 22(46.8) | 0(0.0) |  | 478 | 252(52.7) | 147(30.8) | 79(16.5) | <0.001 |
| Ningxia | 70 | 66(94.3) | 4(5.7) | 0(0.0) |  | 211 | 10(4.7) | 189(89.6) | 12(5.7) | <0.001 |
| **Total** | **423** | **397(93.9)** | **26(6.1)** | **0(0.0)** |  | **1102** | **272(24.7)** | **710(64.4)** | **120(10.9)** | **<0.001** |

**Table A4.7 Proportion of TB patients received adequate diagnostic services**

| **Study sites** | **2015** | | |  | **2018** | | | **P value** |
| --- | --- | --- | --- | --- | --- | --- | --- | --- |
|  | **#Patients** | **#Patients received adequate diagnostic services** | **Proportion (%)** |  | **#Patients** | **#Patients received adequate diagnostic services** | **Proportion (%)** |  |
| Zhejiang | 282 | 227 | 80.5 |  | 420 | 372 | 88.6 | 0.003 |
| Jilin | 268 | 233 | 86.9 |  | 418 | 413 | 98.8 | <0.001 |
| Ningxia | 279 | 247 | 88.5 |  | 347 | 345 | 99.4 | <0.001 |
| **Total** | 829 | 707 | 85.3 |  | 1185 | 1130 | 95.4 | <0.001 |

**Table A4.8 Proportion of smear-negative TB patients received recommended diagnostic services by Guideline**

| **Study sites** | **2015** | | | |  | **2018** | | | | **P value** |
| --- | --- | --- | --- | --- | --- | --- | --- | --- | --- | --- |
|  | **#Patients** | **#Patients received recommended diagnostic services** |  | **Proportion (%)** |  | **#Patients** | **#Patients received recommended diagnostic services** |  | **Proportion (%)** |  |
| **Among asymptomatic pulmonary TB patients** | | | | | | | | | | |
| Zhejiang | 43 | 7 |  | 16.3 |  | 41 | 21 |  | 51.2 | 0.001 |
| Jilin | 8 | 3 |  | 37.5 |  | 7 | 4 |  | 57.1 | 0.447 |
| Ningxia | 4 | 0 |  | 0 |  | 17 | 16 |  | 94.1 | <0.001 |
| **Total** | 55 | 10 |  | 18.2 |  | 65 | 41 |  | 63.1 | <0.001 |
|  |  |  |  |  |  |  |  |  |  |  |
| **Among symptomatic pulmonary TB patients** | | | | | | | | | | |
| Zhejiang | 22 | 21 |  | 95.5 |  | 61 | 47 |  | 77.0 | 0.054 |
| Jilin | 116 | 105 |  | 90.5 |  | 130 | 124 |  | 95.4 | 0.133 |
| Ningxia | 42 | 42 |  | 100 |  | 100 | 95 |  | 95.0 | 0.140 |
| **Total** | 180 | 168 |  | 93.3 |  | 291 | 266 |  | 91.4 | 0.451 |

**Table A4.9 Proportion of drug susceptible TB patients treated by second-line drug (SLD)**

| **Study sites** | **2015** | | | |  | **2018** | | | | **P value** |
| --- | --- | --- | --- | --- | --- | --- | --- | --- | --- | --- |
|  | **#Patients** | **#Patients treated by SLD** |  | **Proportion (%)** |  | **#Patients** | **#Patients treated by SLD** |  | **Proportion (%)** |  |
| Zhejiang | 81 | 12 |  | 14.8 |  | 249 | 80 |  | 32.1 | 0.003 |
| Jilin | 148 | 16 |  | 10.8 |  | 228 | 49 |  | 21.5 | 0.007 |
| Ningxia | 57 | 8 |  | 14 |  | 213 | 30 |  | 14.1 | 0.992 |
| **Total** | **286** | **36** |  | **12.6** |  | **690** | **159** |  | **23** | **<0.001** |

**Table A4.10 Proportion of TB patients receiving recommended follow-up examinations during treatment course in 2015 and 2018, disaggregated by province A) all test B) blood routine test C) liver function test D) liver function test (routine urine test)routine**

| **Study sites** | **2015** | | | |  | **2018** | | | | **P value** |
| --- | --- | --- | --- | --- | --- | --- | --- | --- | --- | --- |
|  | **#Patients** | **#Tests received by patients** | **#Recommended tests** | **Proportion (%)** |  | **#Patients** | **#Tests received by patients** | **#Recommended tests** | **Proportion (%)** |  |
| **All tests** |  |  |  |  |  |  |  |  |  |  |
| Zhejiang | 282 | 561 | 1974 | 28.42 |  | 420 | 2077 | 2940 | 70.65 | <0.001 |
| Jilin | 268 | 344 | 1876 | 18.34 |  | 418 | 1674 | 2926 | 57.21 | <0.001 |
| Ningxia | 279 | 607 | 1953 | 31.08 |  | 347 | 1609 | 2429 | 66.24 | <0.001 |
| **Total** | 829 | 1512 | 5803 | 26.06 |  | 1185 | 5360 | 8295 | 64.62 | <0.001 |
| **Routine blood test** | |  |  |  |  |  |  |  |  |  |
| Zhejiang | 282 | 189 | 564 | 33.51 |  | 420 | 714 | 840 | 85.00 | <0.001 |
| Jilin | 268 | 89 | 536 | 16.60 |  | 418 | 478 | 836 | 57.18 | <0.001 |
| Ningxia | 279 | 181 | 558 | 32.44 |  | 347 | 511 | 694 | 73.63 | <0.001 |
| **Total** | 829 | 459 | 1658 | 27.68 |  | 1185 | 1703 | 2370 | 71.86 | <0.001 |
| **Liver function test** | | |  |  |  |  |  |  |  |  |
| Zhejiang | 282 | 312 | 846 | 36.88 |  | 420 | 1039 | 1260 | 82.46 | <0.001 |
| Jilin | 268 | 160 | 804 | 19.90 |  | 418 | 859 | 1254 | 68.50 | <0.001 |
| Ningxia | 279 | 289 | 837 | 34.53 |  | 347 | 779 | 1041 | 74.83 | <0.001 |
| **Total** | 829 | 761 | 2487 | 30.60 |  | 1185 | 2677 | 3555 | 75.30 | <0.001 |
| **Routine urine test** | |  |  |  |  |  |  |  |  |  |
| Zhejiang | 282 | 60 | 564 | 10.64 |  | 420 | 324 | 840 | 38.57 | <0.001 |
| Jilin | 268 | 95 | 536 | 17.72 |  | 418 | 337 | 836 | 40.31 | <0.001 |
| Ningxia | 279 | 137 | 558 | 24.55 |  | 347 | 319 | 694 | 45.97 | <0.001 |
| **Total** | 829 | 292 | 1658 | 17.61 |  | 1185 | 980 | 2370 | 41.35 | <0.001 |

Note: number of recommended tests = number of patients * 7 (number of recommended tests per patients by guideline)

Recommended tests per patients by guideline: routine blood test *2 (before treatment, first month completion of treatment), liver and renal function test *3 (before treatment, first and second months completion of treatment) and routine urine test *2 (before treatment, first month completion of treatment),

**Table A4.11 Proportion of TB patients receiving recommended follow-up examinations during treatment course in 2015 and 2018, disaggregated by province**

| **Study sites** | **2015** | | | |  | **2018** | | | | **P value** |
| --- | --- | --- | --- | --- | --- | --- | --- | --- | --- | --- |
|  | **#Patients** | **#Number of patients tested** | **# Number of patients not tested** | **Proportion (%)** |  | **#Patients** | **#Number of patients tested** | **# Number of patients not tested** | **Proportion (%)** |  |
| **Routine blood test: before treatment** | | | | |  |  |  |  |  |  |
| Zhejiang | 282 | 0 | 282 | 0.00 |  | 420 | 401 | 19 | 95.48 | <0.001 |
| Jilin | 268 | 81 | 187 | 30.22 |  | 418 | 396 | 22 | 94.74 | <0.001 |
| Ningxia | 279 | 150 | 129 | 53.76 |  | 347 | 334 | 13 | 96.25 | <0.001 |
| **Total** | 829 | 231 | 598 | 27.86 |  | 1185 | 1131 | 54 | 95.44 | <0.001 |
| **Routine blood test: first month completion of treatment** | | | | |  |  |  |  |  |  |
| Zhejiang | 282 | 189 | 93 | 67.02 |  | 420 | 313 | 107 | 74.52 | 0.031 |
| Jilin | 268 | 8 | 260 | 2.99 |  | 418 | 82 | 336 | 19.62 | <0.001 |
| Ningxia | 279 | 31 | 248 | 11.11 |  | 347 | 177 | 170 | 51.01 | <0.001 |
| **Total** | 829 | 228 | 601 | 27.50 |  | 1185 | 572 | 613 | 48.27 | <0.001 |
| **Liver function test: before treatment** | | |  |  |  |  |  |  |  |  |
| Zhejiang | 282 | 0 | 282 | 0.00 |  | 420 | 402 | 18 | 95.71 | <0.001 |
| Jilin | 268 | 65 | 203 | 24.25 |  | 418 | 414 | 4 | 99.04 | <0.001 |
| Ningxia | 279 | 51 | 228 | 18.28 |  | 347 | 338 | 9 | 97.41 | <0.001 |
| **Total** | 829 | 116 | 713 | 13.99 |  | 1185 | 1154 | 31 | 97.38 | <0.001 |
| **Liver function test: first month completion of treatment** | | | |  |  |  |  |  |  |  |
| Zhejiang | 282 | 188 | 94 | 66.67 |  | 420 | 317 | 103 | 75.48 | 0.011 |
| Jilin | 268 | 51 | 217 | 19.03 |  | 418 | 101 | 317 | 24.16 | 0.114 |
| Ningxia | 279 | 117 | 162 | 41.94 |  | 347 | 200 | 147 | 57.64 | <0.001 |
| **Total** | 829 | 356 | 473 | 42.94 |  | 1185 | 618 | 567 | 52.15 | <0.001 |
| **Liver function test: second month completion of treatment** | | | |  |  |  |  |  |  |  |
| Zhejiang | 282 | 124 | 158 | 43.97 |  | 420 | 320 | 100 | 76.19 | <0.001 |
| Jilin | 268 | 44 | 224 | 16.42 |  | 418 | 344 | 74 | 82.30 | <0.001 |
| Ningxia | 279 | 121 | 158 | 43.37 |  | 347 | 241 | 106 | 69.45 | <0.001 |
| **Total** | 829 | 289 | 540 | 34.86 |  | 1185 | 905 | 280 | 76.37 | <0.001 |
| **Routine urine test: before treatment** | | | | |  |  |  |  |  |  |
| Zhejiang | 282 | 0 | 282 | 0.00 |  | 420 | 227 | 193 | 54.05 | <0.001 |
| Jilin | 268 | 95 | 173 | 35.45 |  | 418 | 332 | 86 | 79.43 | <0.001 |
| Ningxia | 279 | 133 | 146 | 47.67 |  | 347 | 258 | 89 | 74.35 | <0.001 |
| **Total** | 829 | 228 | 601 | 27.50 |  | 1185 | 817 | 368 | 68.95 | <0.001 |
| **Routine urine test: first month completion of treatment** | | | | |  |  |  |  |  |  |
| Zhejiang | 282 | 60 | 222 | 21.28 |  | 420 | 97 | 323 | 23.10 | 0.571 |
| Jilin | 268 | 0 | 268 | 0.00 |  | 418 | 5 | 413 | 1.20 | 0.072 |
| Ningxia | 279 | 4 | 275 | 1.43 |  | 347 | 61 | 286 | 17.58 | <0.001 |
| **Total** | 829 | 64 | 765 | 7.72 |  | 1185 | 163 | 1022 | 13.76 | <0.001 |
